# Whole-Genome Identification and Comparative Expression Analysis of Anthocyanin Biosynthetic Genes in *Brassica napus*

**DOI:** 10.3389/fgene.2021.764835

**Published:** 2021-11-18

**Authors:** Dan He, Dawei Zhang, Ting Li, Lili Liu, Dinggang Zhou, Lei Kang, Jinfeng Wu, Zhongsong Liu, Mingli Yan

**Affiliations:** ^1^ School of Life Science, Hunan University of Science and Technology, Xiangtan, China; ^2^ Hunan Key Laboratory of Economic Crops Genetic Improvement and Integrated Utilization, Xiangtan, China; ^3^ Oilseed Research Institute, Hunan Agricultural University, Changsha, China; ^4^ Key Laboratory of Biology and Genetic Improvement of Oil Crops, Ministry of Agriculture and Rural Affairs, Oil Crops Research Institute of Chinese Academy of Agricultural Sciences, Wuhan, China

**Keywords:** anthocyanin biosynthetic genes, *B. napus*, comparative genomic analysis, transcriptome, dynamic accumulation

## Abstract

Anthocyanins contribute to most colors of plants and play protective roles in response to abiotic stresses. *Brassica napus* is widely cultivated worldwide as both an oilseed and a vegetable. However, only several high anthocyanin-containing cultivars have been reported, and the mechanisms of anthocyanin accumulation have not been well-elucidated in *B. napus*. Here, the phenotype, comparative whole-genome identification, and gene expression analysis were performed to investigate the dynamic change of the anthocyanin content and the gene expression patterns of anthocyanin biosynthetic genes (ABGs) in *B. napus*. A total of 152 ABGs were identified in the *B. napus* reference genome. To screen out the critical genes involved in anthocyanin biosynthesis and accumulation, the RNA-seq of young leaves of two *B. napus* lines with purple leaves (PL) or green leaves (GL), and their F_1_ progeny at 41, 91, and 101 days were performed to identify the differentially expressed genes. The comparative expression analysis of these ABGs indicated that the upregulation of *TT8* together with its target genes (such as *DFR*, *ANS*, *UFGT*, and *TT19*) might promote the anthocyanin accumulation in PL at the early developmental stage (41–91 days). While the downregulation of those ABGs and anthocyanin degradation at the late developmental stage (91–101 days) might result in the decrease in anthocyanin accumulation. Our results would enhance the understanding of the regulatory network of anthocyanin dynamic accumulation in *B. napus*.

## Introduction

Anthocyanins are a class of natural pigments responsible for the orange, red to purple, and blue colors of many fruits and vegetables (Buer et al., 2010). Meanwhile, anthocyanins play protective roles by scavenging reactive oxygen species during abiotic stresses and may also serve to reduce cardiovascular risk factors (Butelli et al., 2008; de Pascual–Teresa et al., 2010; Kim et al., 2017).

The anthocyanin biosynthetic genes (ABGs) are well-characterized in a variety of plants, and various structural and regulatory genes play important roles in anthocyanin biosynthesis (Lepiniec et al., 2006; Xu et al., 2013; Liu et al., 2017). The structural genes leading to anthocyanin biosynthesis are usually divided into early biosynthetic genes (EBGs) and late biosynthetic genes (LBGs). EBGs (*chalcone synthase*, *CHS*; *chalcone isomerase*, *CHI*; *flavanone 3-hydroxylase*, *F3H*; *flavonoid 3’-hydroxylase*, *F3’H*) are the common flavonoid pathway genes which are involved in the biosynthesis of all downstream flavonoids. LBGs (*dihydroflavonol 4-reductase*, *DFR*; *anthocyanidin synthase/leucoanthocyanin dioxygenase*, *ANS*/*LDOX*; *uridine diphosphate-glucose: flavonoid 3-O-glucosyltransferase*, *UFGT*) are genes regulating the biosynthesis of anthocyanins (Petroni and Tonelli, 2011; Yan et al., 2014; Liu et al., 2018). The EBGs are regulated by a class of R2R3–MYB transcription factors, while the regulation of LBGs requires a ternary complex of MYB–bHLH–WD40 (MBWs), which binds the promoters of target genes and then activate their transcription, leading to a higher accumulation of the anthocyanin level (Lepiniec et al., 2006; Xu et al., 2014; Kim et al., 2017). In *Arabidopsis thaliana*, the overexpression of MYB regulators, such as *PAP1*, *PAP2*, *MYB113*, and *MYB114* could result in a substantial increase in anthocyanins (Gonzalez et al., 2008). In *Brassica*, transposon insertion and point mutation independently activate the expression of the *BoMYB2* gene in purple cultivars of *Brassica oleracea* (Yan et al., 2019), while a DNA sequence insertion in the first intron greatly reduces the transcription of *BjMYB2* in green cultivars of *Brassica juncea* (Heng et al., 2020a). The overexpression of *BjTT8* and its target genes which are involved in late anthocyanin biosynthesis and transport, account for the increasing levels of anthocyanin accumulation in purple leaves of *B. juncea* (Mushtaq et al., 2016; Heng et al., 2020b; Zhang et al., 2020). However, the expression of *BoMYBL2-1* is inversely correlated to the anthocyanin content, and purple color in *B. oleracea* results from a loss of *BoMYBL2-1* expression (Song et al., 2018). It has been demonstrated that some environmental factors, such as light or temperature, could also influence anthocyanin accumulation by modulating the expression of ABGs (Nguyen et al., 2015; Kim et al., 2017).

*Brassica napus* (AACC, 2*n* = 38), formed by hybridization between the diploid ancestors of *B. rapa* (AA, 2*n* = 20) and *B. oleracea* (CC, 2*n* = 18), is widely cultivated worldwide as both an oilseed and a vegetable (Fujii and Ohmido, 2011; Ton et al., 2020). The purple leaf trait and the mechanisms underlying the anthocyanin accumulation has been widely documented in *B. rapa* and *B. oleracea* (Zhang et al., 2014 and, 2015; He et al., 2016; Xie et al., 2016; Song et al., 2018; Guo et al., 2019; Yan et al., 2019); however, few studies have investigated *Brassica napus* anthocyanin biosynthesis. (Goswami et al., 2018)*.* Previously, the *BnaA.PL1* gene, which confers to purple leaves in *B. napus*, was mapped at the end of chromosome A03 (Li et al., 2016). Transcriptional analyses of a high-anthocyanin resynthesized *B. napus* line by crossing anthocyanin-rich *B. rapa* and *B. oleracea* suggest that *MYB111*, *TT8*, and *TT19* may be responsible for the higher anthocyanin accumulation (Goswami et al., 2018). However, due to the lack of high anthocyanin mutants and genome information, the regulation of the anthocyanin biosynthetic pathway in the economically important plant *B. napus* has not been fully elucidated to date. With the recently released high-quality *B. napus* reference genomes, comparative genomic and transcriptomic analyses are now available for the genome-wide identification of anthocyanin biosynthetic genes responsible for the leaf color (Rousseau–Gueutin et al., 2020; Song et al., 2020; Mohd Saad et al., 2021). Herein, the anthocyanin contents of two *B. napus* lines with purple leaves or green leaves, and their F_1_ progeny at different developmental stages were measured to investigate the dynamic change trend. A combination of comparative genomic and transcriptomic analysis was performed to identify the ABGs in the *B. napus* genome and to investigate the gene expression patterns of these ABGs in leaves with different colors. Taken together, our results provide a comprehensive perspective of the transcriptional regulation of the anthocyanin biosynthetic pathway in *B. napus*.

## Materials and Methods

### Plant Materials

One *B. napus* inbred lines which exhibited purple leaves (abbreviated as PL) at the seedling stage and ZS11 (Zhongshuang 11) which showed green leaves (abbreviated as GL), as well as the F_1_ progeny by a cross between these two lines were used in this study. Seeds of PL, GL, and F_1_ were sown directly on September 30th, 2020, and three seedlings of each were maintained in the same pot (43 cm length × 40 cm wide × 25 cm high) to minimize the developmental or environmental effects. All the plants were grown under natural lighting in a greenhouse located at the Hunan University of Science and Technology (27°91′05″N, 112°92′60′'E, China). Considering that the leaf color of PL showed a dynamic change from light purple to dark purple and then to green, the fresh leaves at the seedling stage (<101 days after sowing) and bolting stage (150 days after sowing) were collected and stored at −80°C.

### Analysis of Anthocyanins

The total anthocyanin content was measured using the method described previously (Li et al., 2016; Zhang et al., 2020). In brief, the young leaves PL, GL, and F_1_ with three replicates were ground into powder after low-temperature freeze drying (SJIA-10N, Ningbo, China). Then 0.1 g powder was mixed with 1 ml of 95% ethanol: 1.5 M HCl (85:15, v/v) for 20 h at 4°C in the dark with moderate shaking. The aqueous phase was collected after centrifuging at 12,000 ×g for 10 min, and the absorbance was subsequently measured at 530 nm using a Cary 60 UV-Vis (Agilent Technologies, Palo Alto, United States).

### Identification of Anthocyanin Biosynthetic Genes in *B. napus*

Previously, the ABGs in *B. rapa* were well-characterized based on the syntenic analysis results between *B. rapa* and *A. thaliana* (Guo et al., 2014)*.* Following the method provided by Cheng et al. (2012) and Guo et al. (2014 and 2019), syntenic analysis across *B. napus*, *A. thaliana*, *B. rapa*, and *B. oleracea* was performed, and ABGs were identified using SynOrths (http://brassicadb.cn/#/Download/) with the same parameters (NumQ = 20, NumR = 100, RatioQR = 0.2). Gene pairs that are the best hits or with Blastp e-values <1E^−20^ were selected for further analysis. The reference genome of *A. thaliana* was downloaded from BRAD (http://brassicadb.cn), as described before. The *B. oleracea* (genotype HDEM) and *B. rapa* (genotype Z1) reference genomes were obtained from https://www.genoscope.cns.fr/externe/plants (Rousseau-Gueutin et al., 2020). The *B. napus* (genotype ZS11) reference genome was obtained from http://cbi.hzau.edu.cn/bnapus/(Song et al., 2020), respectively. Integrative software TBtools was used for determining the chromosomal locations of ABGs of *B. napus* (Chen et al., 2020).

### RNA Extraction and RNA-Seq

The entire of fully expanded young leaves from PL, GL, and F_1_ with three biological replicates at 41, 91, and 101 days after sowing were collected for further RNA-seq, respectively. The total RNA of samples was extracted using the RNA Easy Fast Plant Tissue Kit (TIANGEN, Beijing, China) according to a standard protocol. After that, 1.5% agarose gel, a NanoPhotometer® spectrophotometer (IMPLEN, CA, United States), and a Bioanalyzer 2,100 system (Agilent Technologies, CA, United States) were also used to check the purity and integrity of RNA. Samples with RIN scores≥7.5 and OD260/280 = 1.8–2.2 were utilized for further sequencing. Sequencing libraries were generated using the NEBNext® UltraTM RNA Library Prep Kit for Illumina® (NEB, United States), following the manufacturer’s recommendations. The libraries were sequenced on an Illumina NovaSeq 6,000 platform and 150-bp paired-end reads were generated by Shanghai Majorbio Bio-pharm Technology Co., Ltd.

### Reads Filtering and Mapping

After the high-throughput sequencing, the low-quality reads which contained adapters and ploy-N were trimmed using Trimmomatic. Then, the clean reads were mapped to the high-quality *B. napus* (genotype ZS11; http://cbi.hzau.edu.cn/bnapus/) reference genomes using TopHat2 (http://tophat.cbcb.umd.edu/) with the default parameters. Software RSEM was used for read counting, and DESeq2 was used for differential expression analysis. Genes with adjusted *p*-values < 0.05 were classified as differentially expressed. The expression levels of individual genes were also quantified using the transcripts per million (TPM) method. The KEGG enrichment analysis of differentially expressed genes was implemented using the free online platform of Majorbio Cloud Platform (www.majorbio.com). KEGG terms with corrected *p*-values less than 0.05 were considered to be significantly enriched.

### Validation of the Expression Data by qRT-PCR

The extracted RNA was also used for qRT–PCR. First-strand cDNA synthesis was performed using a Tsingke Goldenstar RT6 cDNA Synthesis Kit ver.2 (Tsingke, Beijing, China). Differentially expressed ABGs were selected, and specific primers were designed for qRT–PCR using Oligo 7 and NCBI Primer-BLAST (https://www.ncbi.nlm. nih. gov/tools/primer-blast/). The *β-actin* gene (GenBank accession No. AF111812) was used as an internal control, and SYBR Premix Ex TaqII with a Bio-Rad CFX96 Real-Time Detection System was used as described previously (Zhang et al., 2018).

## Results

### Phenotypic Characterization of Purple Leaves and Green Leaves

**FIGURE 1:**
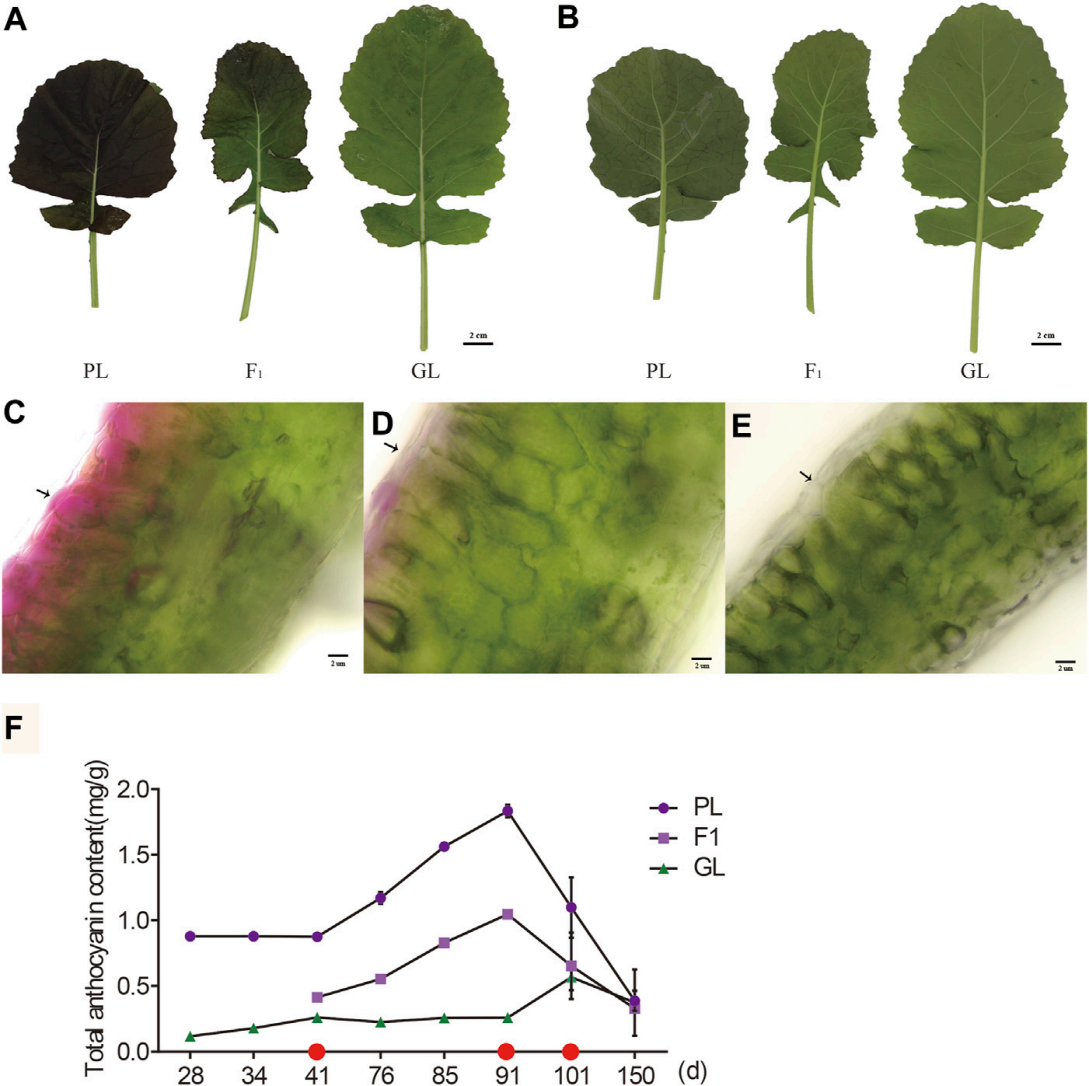
Phenotypes of *B. napus* with different leaf colors. The upper **(A)** and lower epidermis **(B)** of PL, F_1_, and GL (scale bar = 2 cm). Transverse section of leaf from PL **(C)**, F_1_ **(D)**, and GL **(E)** with different colors (scale bar = 2 μm). **(F)** The content of total anthocyanin accumulation at different stages. Values represent means ± SD from three biological replicates. Red dots in the x-axis indicate days for RNA-seq sampling.

During the seedling stage, the PL displayed purple pigments in the surface and thin vein of leaves, while the F_1_ only showed faint purple in the leaf surface (Figures 1A,B). The microscopic observation of sections revealed that the purple pigments were mainly accumulated in the adaxial epidermis and subepidermal cells of PL leaves, while it decreased in the top cell layer of F_1_ (Figures 1C,D). In contrast, GL with common green color only exhibited green chlorophyll layers in leaves (Figure 1E).

Although the average anthocyanin contents in PL (1.09 mg/kg DW) were about 3.9-fold higher than those in GL (0.28 mg/kg DW, *p* < 0.05, paired Students’ *t*-test), they varied at different developmental stages in the leaves of PL (Figure 1F). During the seedling stage (<101 days), the total anthocyanin contents of PL were stable at initial weeks (0.88 mg/kg DW, 28–41 days after sowing) and increased after that (76–91 days) but dropped dramatically from 1.83 mg/kg (91 days) to 1.10 mg/kg (101 days). No significant difference (*p* > 0.05, *t*-test) was observed between PL (0.39 mg/kg) and GL (0.37 mg/kg) at the bolting stage (150 days), which was correlated with the color change from purple to green in the leaves of PL. The average anthocyanin contents in F_1_ (0.64 mg/kg DW) were higher than those in GL but significantly lower than those in PL (*p* < 0.05, paired *t*-test), suggesting that incompletely dominant gene or genes might control the purple leaf trait in *B. napus*.

### Genome-Wide Identification of Anthocyanin Biosynthetic Genes in *B. napus*

Based on the method from a previous study (Guo et al., 2019; Zhang et al., 2020), a total of 74, 93, and 152 genes were identified to be ABGs in *B. rapa*, *B. oleracea*, and *B. napus* (Supplementary Table S1). As compared with 41 ABGs in *A. thaliana*, *FLS2*, *FLS4*, *FLS5*, *FLS6*, *CPC*, *MYB111*, *MYB113*, and *MYB114* have not been identified in *B. rapa*, *B. oleracea*, and *B. napus*; these observations suggested that these genes were lost following the whole-genome triplication*.* Among the 152 genes identified in *B. napus*, 98 were classified into structural genes, 50 were regulatory genes, and 4 were transport genes. The number of these three types of genes was quadrupled as compared with *A. thaliana*, which may be caused by whole-genome triplication and subsequent allopolyploidization. Using the total *A. thaliana* and *B. napus* genes as references, the 152 ABGs represent 0.15% of the 100,919 annotated *B. napus* genes, which was similar to that in *A. thaliana* (0.15%). Meanwhile, the overall expansion levels of structural, regulatory, and transport genes also exhibited no significant difference to the whole genome (Table 1, chi-squared test, *p* > 0.05).

**TABLE 1:** Comparison of the number of ABGs in *Arabidopsis thaliana*, *Brassica rapa*, *Brassica oleracea*, and *Brassica napus*.

	*A. thaliana*	*B. rapa*	*B. oleracea*	*B. napus*	*p*-value
Structural genes	24	50	63	98	0.73
Regulatory genes	16	22	28	50	0.67
Transport genes	1	2	2	4	0.83
Total	41	74	93	152	1.00

The proportion of total A. thaliana and B. napus genes was used as a background to calculate the *p*-value using the chi-squared test. A *p*-value greater than 0.05 indicates that the proportion of ABGs between A. thaliana and B. napus was not significantly different from the background.

**FIGURE 2:**
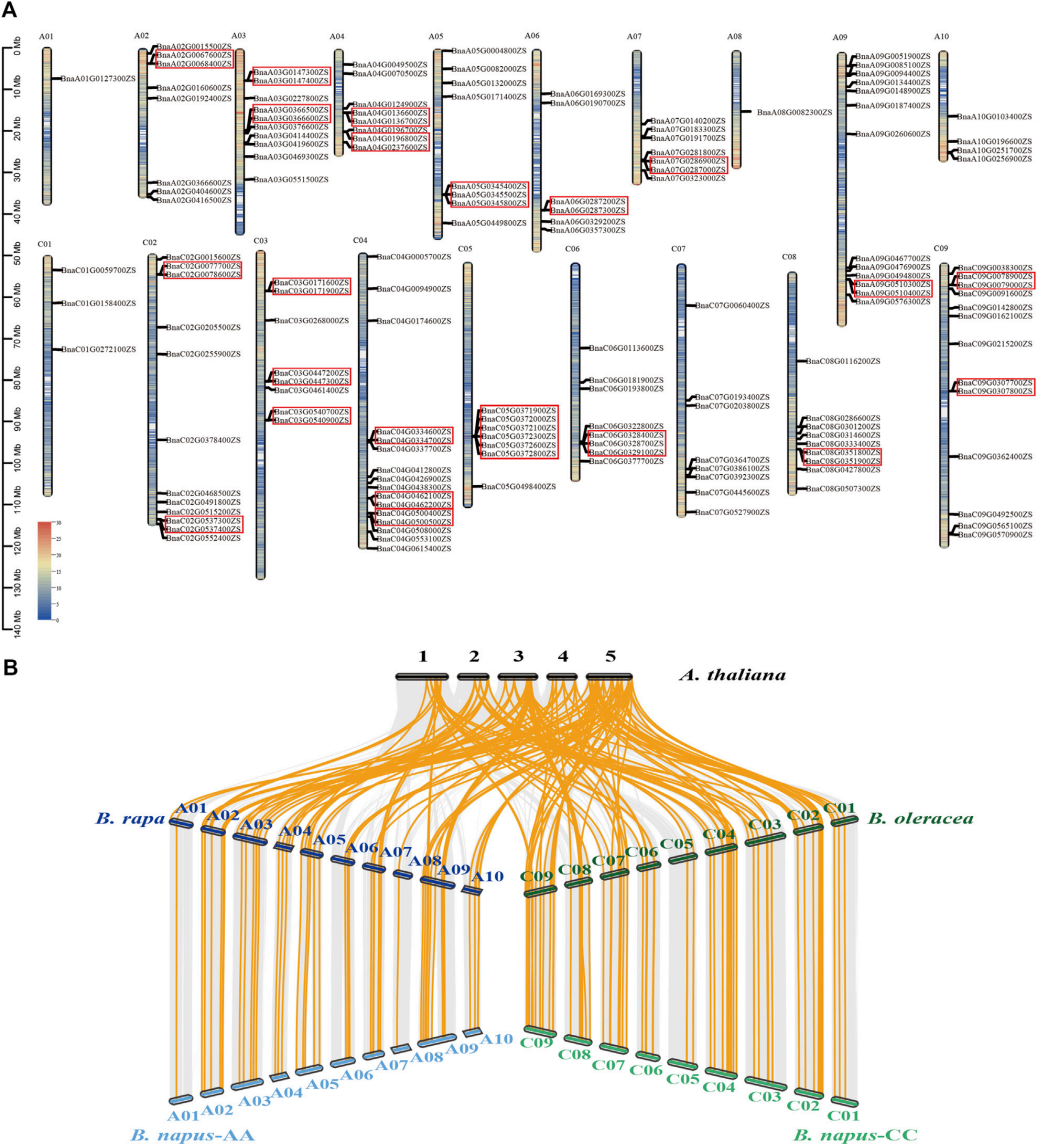
Distribution and syntenic analysis of ABGs in *Brassica napus*. **(A)** The color on the chromosome indicates the gene density of all the genes. Redder color indicates the higher gene density, while the bluer color indicates lower gene density. The red boxes indicate tandem duplications. **(B)** The orange lines indicate the ABGs exhibited syntenic between *A. thaliana*, *B. rapa*, *B. oleracea*, and *B. napus*.

Among these 152 ABGs, 150 were anchored to the 19 chromosomes of *B. napus*, in which 66 genes were distributed on the A subgenome and other 84 genes were located on the C subgenome, ranging from 1 to 16 on each chromosome (Figure 2A). Among the 150 anchored ABGs, 33.3% (50/150) were originated from tandem duplication events. The 50 tandemly duplicated genes could be divided into 22 groups, with 9 groups on the A subgenome (19/50) and 13 groups on the C subgenome (31/50), of which 19 groups contained two genes each, two groups contained 3 genes each, and 1 group contained 6 genes each. Except for one set of tandemly duplicated gene on the chromosome A02 and two set of tandemly duplicated genes on chromosome C02 and chromosome C05, respectively, all the tandemly duplicated genes had homologous copies both in A and C subgenomes. These indicated that most tandem duplication events occurred before polyploidization. The syntenic analysis of ABGs among *A. thaliana*, *B. rapa*, *B. oleracea*, and *B. napus* showed that the whole-genome triplication of *Brassica* and subsequent allopolyploidization contributed to the expansion of ABGs in *B. napus* (Figure 2B)*.* Although some genes were lost as a result of gene fractionation that occurred following the whole-genome triplication, a large proportion of ABGs exhibited syntenic between *B. napus* and *B. rapa*/*B. oleracea*.

### Comparative Transcriptome Analysis of *B. napus* With Different Leaf Colors

To investigate the molecular mechanisms of anthocyanin accumulation, young leaves with three biological replicates at three different developmental stages (41, 91, 101 days) from GL, F_1_, and PL were collected for library construction (3 × 3 × 3 = 27 libraries) and high-throughput RNA sequencing (Supplementary Table S2). In summary, about 127.0 G clean reads were obtained after filtering, and more than 90% of them can map on the *B. napus* reference genome released recently (Song et al., 2020). Gene expression levels were calculated using the TPM methods, and gene expression correlation showed good consistence between replications (Supplementary Figure S1).

**FIGURE 3:**
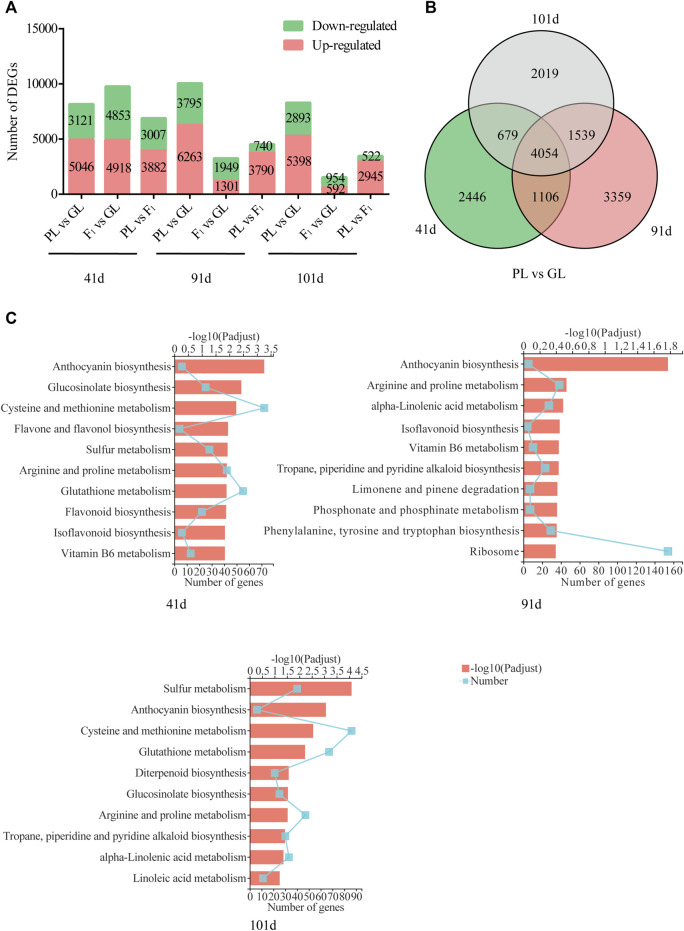
Summary of differentially expressed genes between GL and PL. **(A)** The number of DEGs among GL, F_1_, and PL. **(B)** Venn diagrams of differentially expressed genes between PL and GL shared at different developmental stages. **(C)** The KEGG enrichment of DEGs between PL and GL at different developmental stages.

The comparative transcriptome analysis of *B. napus* among GL, F_1_, and PL showed that the number of DEGs was varied in different comparisons at different developmental stages (Figure 3A). The highest number of DEGs was found between PL and GL at 91 days when PL showed highest anthocyanin content, for 6,263 genes were upregulated and 3,795 genes were downregulated. While the number of DEGs were decreased between F_1_ and the parental lines (F_1_ vs GL and PL vs F_1_) with the development of plants, and the lowest number of DEGs was found between F_1_ and GL at 101 days when the anthocyanin content showed no significant difference. About 8,167, 10,058, and 8,291 genes were differentially expressed between PL and GL at 41, 91, and 101 days after sowing, and 4,054 of them were shared in the aforementioned comparisons (Figure 3B). The KEGG analysis of those DEGs between PL and GL suggested that the genes involved in anthocyanin biosynthesis were significantly enriched at 41, 91, and 101 days (Figure 3C).

### The Expression Analysis of Genes in the Anthocyanin Biosynthetic Pathway in the Leaves of *B. napus*

**FIGURE 4:**
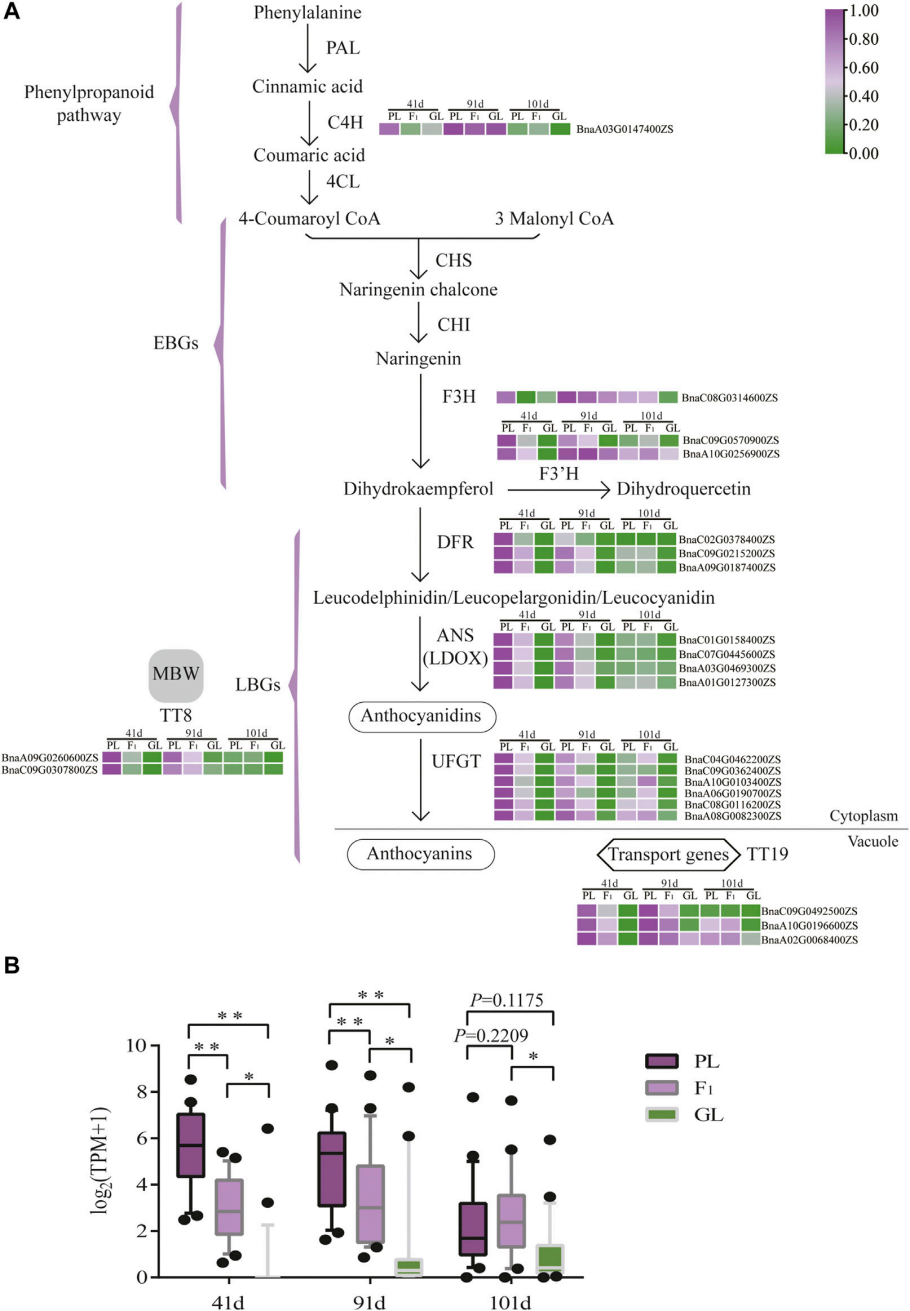
Anthocyanin biosynthetic pathway and expression patterns of ABGs in *B. napus.* **(A)** The expression level of ABGs which are differentially expressed between PL and GL is shown in the pathway. Purple and green colors are used to represent high to low expression levels, respectively. The color scale corresponds to the mean-centered log2-transformed average TPM values from three replicates. **(B)** The overview of ABG expression, which was differentially expressed at different stages. Asterisks indicate statistical differences between each other (two-tailed Student’s *t*-test, ** indicates *p* < 0.01; * indicates *p* < 0.05).

To identify key genes responsible for leaf color variation in *B. napus*, the expression patterns of ABGs were investigated. Among the 152 ABGs, the expression levels of 57 genes were extremely low (average TPM value < 1) or undetected (Supplementary Table S3). Of the other 95 expressed ABGs, 22 genes were differentially expressed between PL and GL. These 22 DEGs are equally distributed in the A subgenome (11/22) and C subgenome (11/22), while the average expression level of the 11 DEGs in C subgenome was higher than that in the A subgenome (*p* < 0.05, two-tailed Student’s *t*-test; Figure 4B). Based on these results, the expressed levels of DEGs in the anthocyanin biosynthetic pathway from different leaf color samples at different stages were demonstrated (Figure 4). The majority of upstream genes in the anthocyanin biosynthetic pathway (45/46 in the phenylpropanoid pathway and 32/35 in EBGs) were not differentially expressed, implying that these genes might not play important roles in anthocyanin accumulation differences. While these genes which are involved in LBGs, such as *DFR* (3/3), *ANS* (3/3), and *UFGT* (6/9), as well as *TT19* (3/4) and *TT8* (2/3) were significantly upregulated in PL, as compared with GL at 41 and 91 days after sowing (Supplementary Table S3, Figure 4A). As to F_1_, the overall expression level of 22 ABGs was significantly higher than that in GL at 41, 91, and 101 days but lower than that in PL at 41 and 91 days (*p* < 0.05, two-tailed Student’s *t*-test; Figure 4B). Except for one *C4H* gene (BnaA03G0147400ZS), all the other genes (21/22) involved in early or late anthocyanin biosynthetic exhibited the similar trend of expression level PL > F_1_>GL at 41 and 91 days (Figure 4A). Quantitative RT-PCR results also confirmed the overexpression of these genes in PL and F_1_, suggesting that these genes might be the essential factors for anthocyanin accumulation at the early stage (Supplementary Figure S2, Supplementary Table S4). It is also worthy to note that the expression levels of most upregulated (*p* < 0.01, paired *t*-test) genes in PL at 41 and 91 days were decreased due to the similar levels of GL (*p* = 0.1175, paired *t*-test) at 101 days, which was consistent with the decrease in anthocyanin contents and the leaf color phenotype in PL (Figure 4B and Figure 1F).

To further understand the relationship between expression patterns of the 22 differentially expressed ABGs and anthocyanin accumulation, correlation coefficients were calculated (Supplementary Figure S3). Significant correlations (*p* < 0.05, Pearson correlation coefficient) between expression and anthocyanin contents were observed in *UFGT* (BnaA08G0082300ZS, *r* = 0.60), *TT8* (BnaA09G0260600ZS, *r* = 0.62), *TT19* (BnaC09G0492500ZS, *r* = 0.76; BnaA10G0196600ZS, *r* = 0.82; BnaA02G0068400ZS *r* = 0.72), *F3H* (BnaC08G0314600ZS, *r* = 0.79), and *F3 'H* (BnaA10G0256900ZS, *r* = 0.64).

## Discussion

In plants, anthocyanins not only confer colorful appearances but also play protective roles against various abiotic and biotic stresses; thus, the mechanisms underlying anthocyanin accumulation have been widely studied in many plant systems (Liu et al., 2015; Xu et al., 2015; Zhang et al., 2017; Castillejo et al., 2020; Yan et al., 2011). In *Brassica*, the drastic differences in anthocyanin accumulation and distribution could arise from cultivar and genetic discrepancies. Herein, our results found that the anthocyanins were mainly accumulated in the adaxial epidermis of PL leaves, which was consistent with the findings in *B. rapa* but inconsistent with that in *B. juncea* and *B. oleracea* (Zhang et al., 2015; He et al., 2016; Mushtaq et al., 2016; Heng et al., 2020b; Zhang et al., 2020)*.* The anthocyanin contents in PL were significantly higher than those in GL but varied at different developmental stages. The highest content in PL was found 91 days after sowing at the seedling stage but dropped dramatically to the same level of GL after 150 days at the bolting stage (Figure 1). The dynamic changes in the anthocyanin content were correlated with the phenotype change from purple to green in the leaves, suggesting that anthocyanin accumulation was a response to leaf color in *B. napus* and influenced by the environmental and developmental signals. Previous studies demonstrated that anthocyanin biosynthesis could be regulated by many environmental factors, such as light, temperature, and various types of biotic and abiotic stresses, via a network comprising many transcription factors (Nguyen et al., 2015; He et al., 2016; Wang et al., 2016; Kim et al., 2017). For example, flavonoid biosynthesis might be induced to accumulate anthocyanin in the red-leaved accessions of ornamental kale (*B. oleracea* var*. acephala*), when plants were under low temperature (Guo et al., 2019). Consistent with those studies, the anthocyanin content in PL also increased with the decrease in temperature (from 22°C at 41 days to 7°C at 91 days) and decreased with the increase in temperature (7°C at 91 days to 11°C at 101 days). Thus, we hypothesized that the purple color intensity in *B. napus* leaves, which was caused by differences in total anthocyanin content, could be regulated by a diverse array of exogenous and endogenous environmental factors.

A previous study suggested that 73 ABGs in *B. rapa*, 81 genes in *B. oleracea*, and 129 genes in *B. juncea* were found as orthologs of 41 ABGs in *A. thaliana*, respectively (Guo et al*.* 2014; Guo et al*.* 2019; Zhang et al*.* 2020). In the present study, a total of 74, 93, and 152 genes were identified to be ABGs in *B. rapa*, *B. oleracea*, and *B. napus* using the reference genome released recently (Rousseau-Gueutin et al., 2020; Song et al., 2020; Supplementary Table S1). The *B. napus* genome has undergone a whole-genome triplication of *Brassica* and recent allopolyploidy between *B. rapa* and *B. oleracea* because of its divergence from the *A. thaliana*; thus, the copy number of ABGs in *B. napus* has been expanded following the whole-genome duplication events (Chalhoub et al., 2014)*.* However, these events were followed by diploidization that involved substantial genome reshuffling and gene losses, which might lead to different copy number retention of the homologous genes of *A. thaliana* (Wang et al., 2011; Liu et al., 2014). The comparative and syntenic analyses of ABGs between *Brassica* and *A. thaliana* genome suggested that gene losses have mainly occurred following the whole-genome triplication between *B. rapa/B. oleracea* and *A. thaliana*, rather than the allopolyploidy between *B. napus* and *B. rapa/B. oleracea* (Figure 2), which was in accordance with the findings in *B. juncea* (Zhang et al., 2020).

**FIGURE 5:**
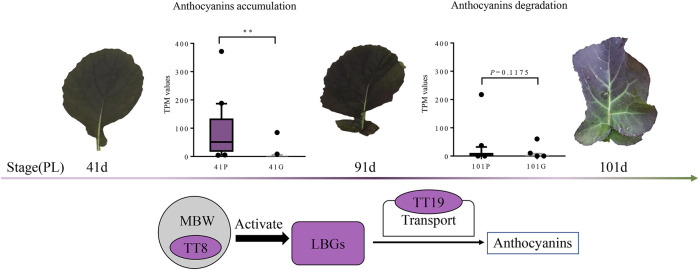
Model of anthocyanins accumulation in *Brassica napus*. The *TT8*, *TT19*, and LBGs are significantly expressed in PL compared with those in GL from 41 to 91 days, (*p* < 0.01). At these developmental stages, anthocyanin contents increase, and the purple pigments are accumulated in the adaxial epidermis and subepidermal cells. The expression levels of these genes are decreased in PL and exhibited no significant difference between that of GL from 91 to 101 days, (*p* = 0.1175). The anthocyanin degradation at this developmental stage might reduce the total anthocyanin accumulation and result in the leaves to gradually turn to green. Circles filled with purple color indicate upregulated genes expressed in PL at 41 and 91 days.

The comparative transcriptome analysis of *B. napus* with different leaf colors found that about 22 ABGs were upregulated in purple leaves relative to green leaves. As compared with two parental lines, the expression level of 21 ABGs in F_1_ was also higher than that in GL but lower than that in PL at 41 and 91 days, which was in accordance with the phenotype of the intermediate anthocyanin content (Figure 1F, Figure 4). Among the 22 upregulated genes, late ABGs (*DFR*, *ANS*, and *UFGT*), positive regulatory genes (*TT8*), and transport genes (*TT19*) exhibited significant correlations between their expression levels and anthocyanin contents, indicating that these genes might play important roles in anthocyanin accumulation differences (Supplementary Figure S3). The overexpression of genes involved in late anthocyanin biosynthesis, positive regulatory as well as transport genes, is usually responsible for increased levels of anthocyanin accumulation in plants (Sun et al., 2012; Zhang et al., 2015; He et al., 2016; Kim et al., 2017; Goswami et al., 2018; Heng et al., 2020b). The ectopic expression of *AtDFR* resulted in increased accumulation of anthocyanins in *B. napus* plants (Kim et al., 2017). While *AtTT19* functioned as a carrier to transport anthocyanin from the cytosol to tonoplasts, and the *tt19* mutant barely accumulates anthocyanins (Sun et al., 2012). In vegetative tissues, *DFR*, *ANS*, *UFGT*, and *TT19* were the direct targets of the MBW ternary complex, and the expression of these genes relied on the transcriptional activity of R2R3-MYB, bHLH, and WDR proteins (Xu et al., 2014). As a central component in the MBW complex, TT8 was a key regulator and could activate the anthocyanin biosynthesis in *B. rapa*, *B. oleracea*, and *B. juncea* (He et al., 2016; Li et al., 2016; Guo et al., 2019; Heng et al., 2020b). Consistent with previous studies, *TT8* and *TT19* displayed higher regulation levels in PL at 41 and 91 days, as well as significantly high correlation between the expression and anthocyanin contents at different stages, suggesting that *TT8* and *TT19* might play vital roles in anthocyanin accumulation (Figures 4,5). Hence, it is speculated that the upregulation of *TT8* together with its target genes (such as *DFR*, *ANS*, *UFGT*, and *TT19*) might promote the anthocyanin accumulation in *B. napus* under low temperature at the early stage (41–91 days). Notably, the expression levels of most upregulated genes in PL at 91 days were decreased to similar levels of GL at 101 days, which was consistent with the decrease in anthocyanin contents in PL (Figure 5). The downregulation of those ABGs and time-consuming anthocyanin degradation under higher temperature at the late developmental stage (91–101 days) might reduce the total anthocyanin accumulation, leading to a color change from purple to green in the leaves of PL.

## Conclusion

We found that the anthocyanins were mainly accumulated in the adaxial epidermis and could be influenced by the environmental and developmental signals. A total of 152 ABGs were identified in the *B. napus* genome, and they have been expanded during the whole-genome duplication events, but some of the genes were lost following genome diploidization. The expression patterns of these ABGs in different leaf types indicated that the upregulation of *TT8* together with its target genes (such as *DFR*, *ANS*, *UFGT*, and *TT19*) might promote the anthocyanin accumulation in *B. napus* at early stages (41–91 days), while the downregulation of those ABGs and anthocyanin degradation at late developmental stages (91–101 days) might result in the reduction of anthocyanin accumulation. Taken together, our results enhance the understanding of the genetic mechanisms and regulatory network of anthocyanin accumulation in *B. napus.*

## Data Availability

The datasets presented in this study can be found in online repositories. The names of the repository/repositories and accession number(s) can be found at https://ngdc.cncb.ac.cn/gsa/, CRA004724.
