# Supplementary figures and images for: Whole-Genome Identification and Comparative Expression Analysis of Anthocyanin Biosynthetic Genes in Brassica napus
Source: Front Genet. 2021 Nov 18;12:764835. doi: 10.3389/fgene.2021.764835 (PMC8636775; doi:10.3389/fgene.2021.764835)

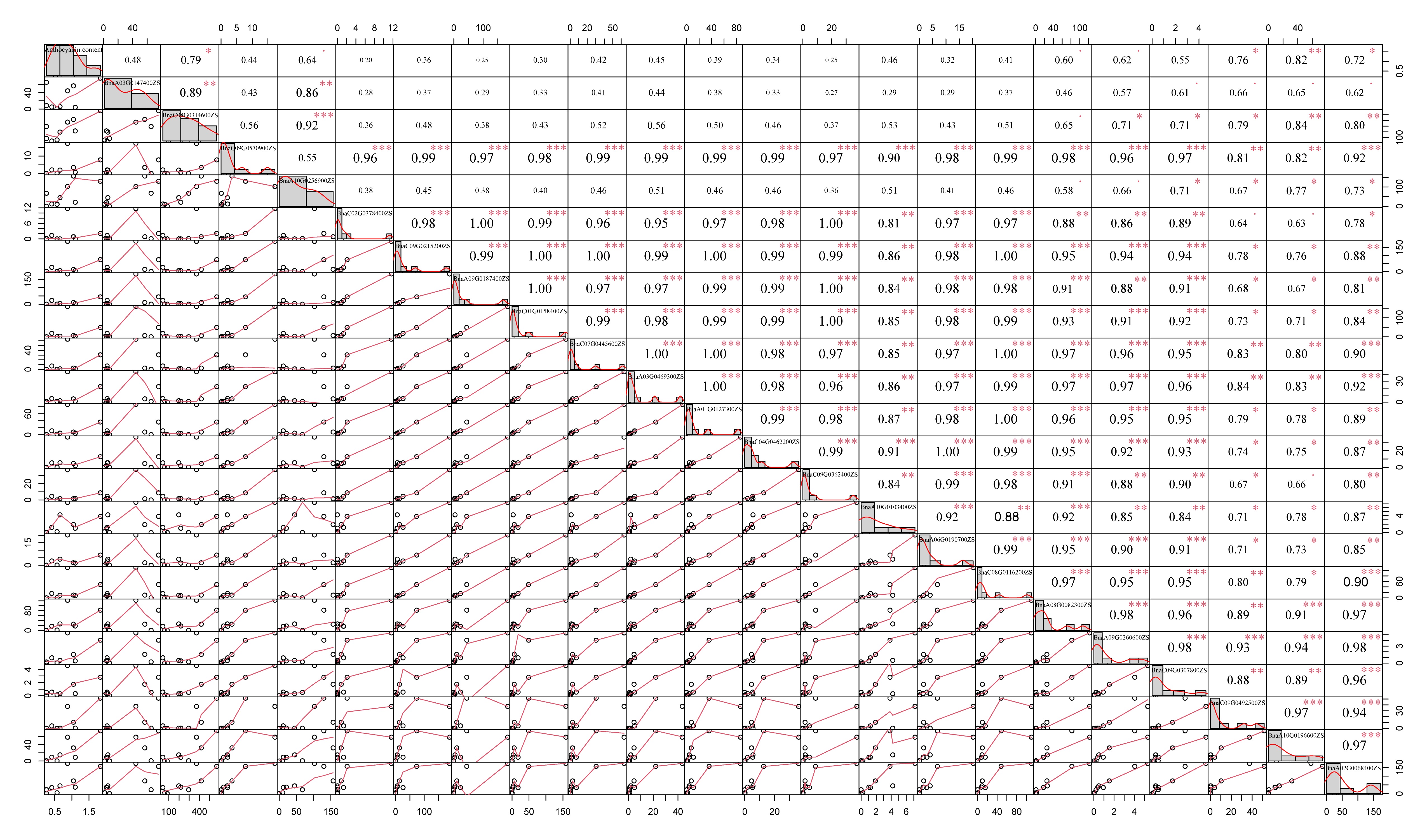

Supplement: Supplementary file 2 [file Image3.JPEG]

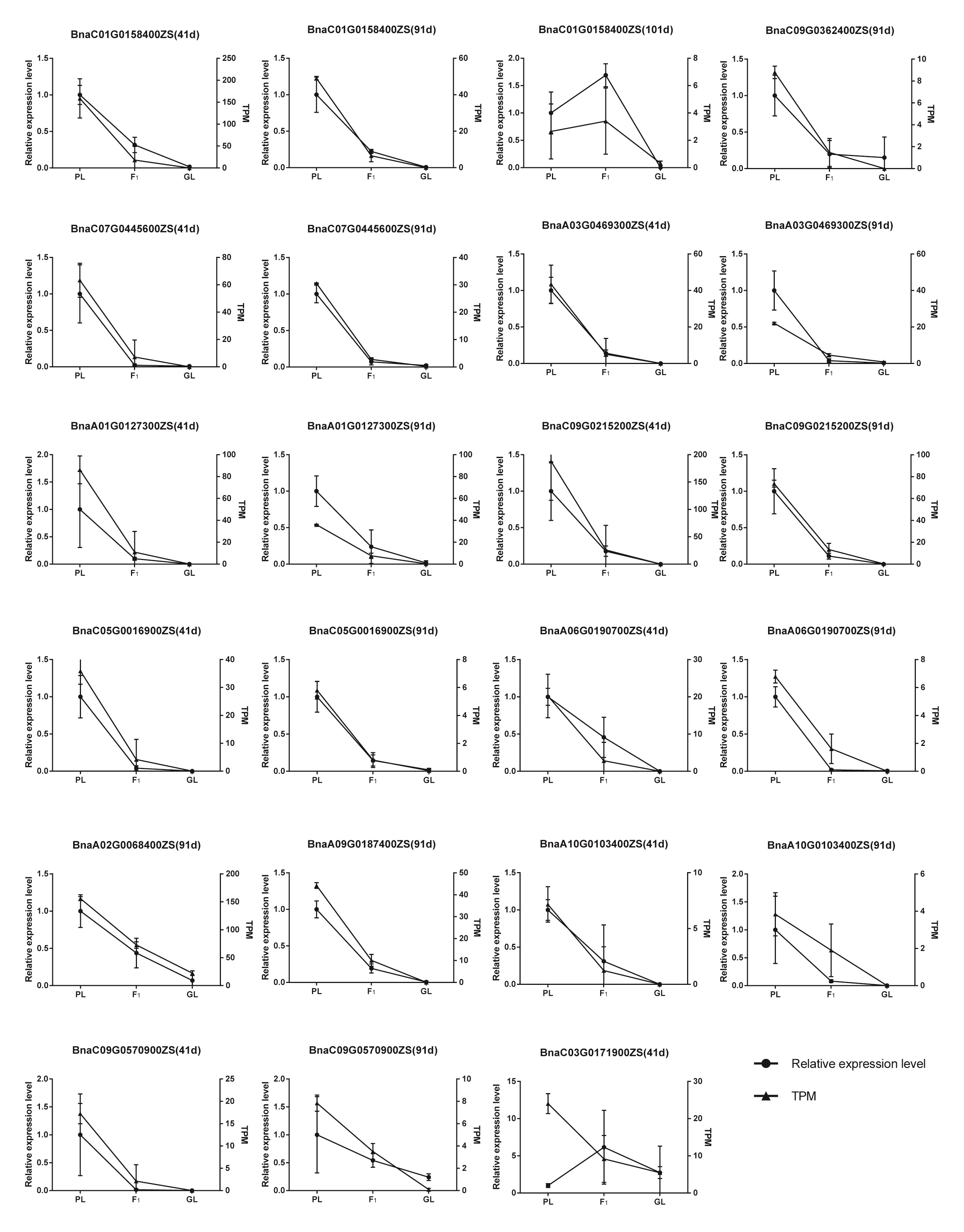

Supplement: Supplementary file 5 [file Image2.JPEG]

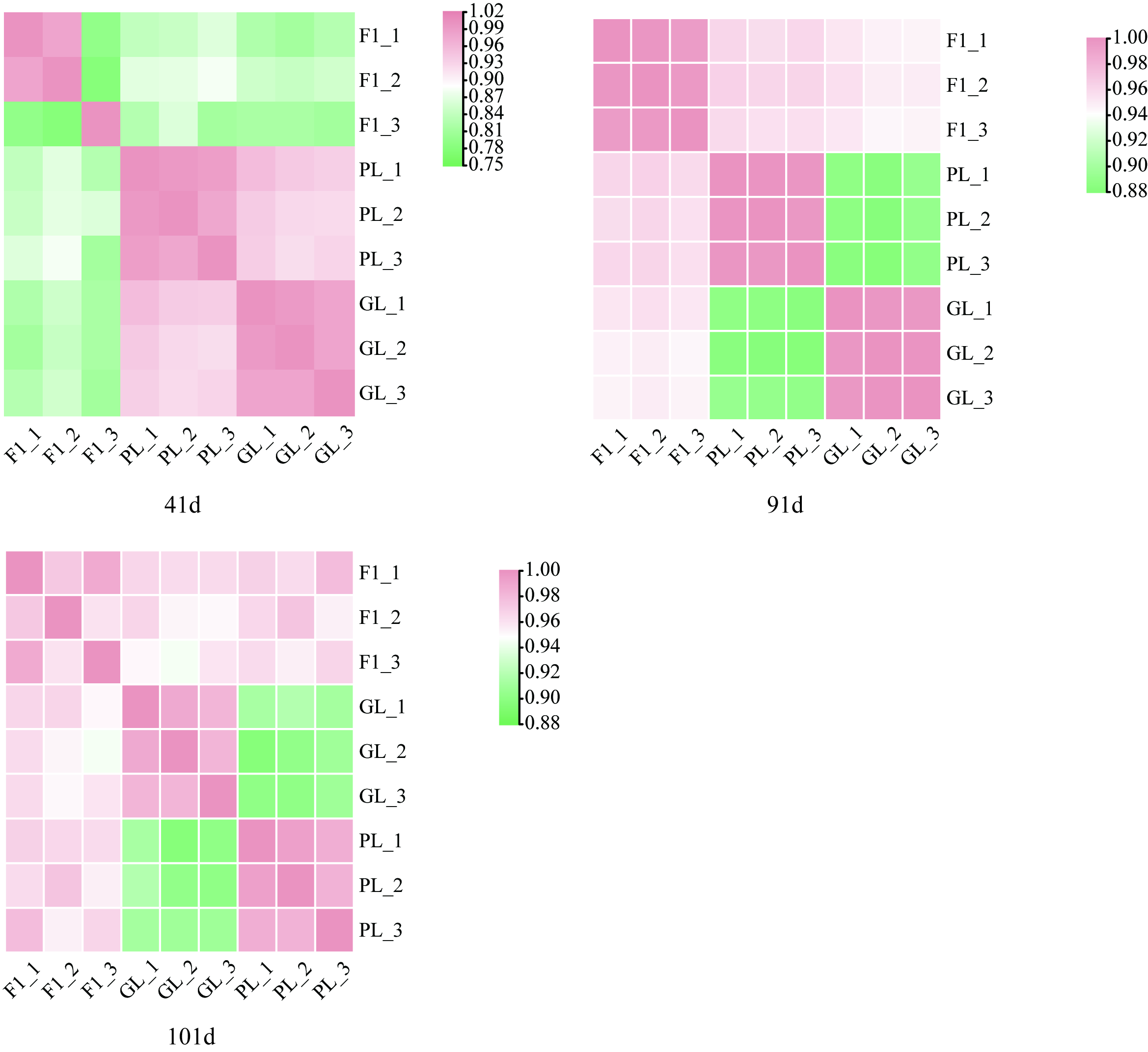

Supplement: Supplementary file 6 [file Image1.TIF]
