# Supplementary material for: Whole-Genome Identification and Comparative Expression Analysis of Anthocyanin Biosynthetic Genes in Brassica napus
Source: Front Genet. 2021 Nov 18;12:764835. doi: 10.3389/fgene.2021.764835 (PMC8636775; doi:10.3389/fgene.2021.764835)
Supplement: Supplementary file 4 [file Table1.DOCX]

Table S1 Anthocyanin biosynthetic genes identified in *B.rapa, B.oleracea* and *B.napus*

| Gene | | | | *A. thaliana* | *B.rapa* | *B.oleracea* | *B.napus* |
| --- | --- | --- | --- | --- | --- | --- | --- |
| Structural | Biosynthetic |  | *PAL1* | AT2G37040 | BraA04g18313Z | BolC4g22683H | BnaC04G0094900ZS |
| genes | genes in |  |  |  | BraA05g19846Z | BolC4g28059H | BnaA04G0237600ZS |
|  | phenylpropanoid |  |  |  |  |  | BnaC04G0553100ZS |
|  | pathway |  |  |  |  |  | BnaA05G0082000ZS |
|  |  |  | *PAL2* | AT3G53260 | BraA09g40092Z | BolC8g50456H | BnaC04G0334600ZS |
|  |  |  |  |  | BraA07g29836Z | BolC6g37294H | BnaA04G0070500ZS |
|  |  |  |  |  | BraA04g16324Z | BolC4g25539H | BnaC06G0181900ZS |
|  |  |  |  |  |  | BolC4g25500H | BnaC04G0334700ZS |
|  |  |  |  |  |  |  | BnaA09G0494800ZS |
|  |  |  |  |  |  |  | BnaC04G0337700ZS |
|  |  |  |  |  |  |  | BnaC08G0333400ZS |
|  |  |  |  |  |  |  | BnaA07G0183300ZS |
|  |  |  | *PAL3* | AT5G04230 | BraA02g04805Z | BolC4g26629H | BnaA02G0015500ZS |
|  |  |  |  |  | BraA04g17220Z | BolC2g06142H | BnaA04G0136600ZS |
|  |  |  |  |  | BraA04g17221Z |  | BnaA04G0136700ZS |
|  |  |  |  |  |  |  | BnaC04G0426900ZS |
|  |  |  | *PAL4* | AT3G10340 | BraA04g17217Z | BolC5g34439H | BnaA05G0449800ZS |
|  |  |  |  |  | BraA05g22672Z |  | BnaC02G0015600ZS |
|  |  |  |  |  |  |  | BnaC05G0498400ZS |
|  |  |  | *C4H* | AT2G30490 | BraA05g20423Z | BolC4g27445H | BnaC03G0171600ZS |
|  |  |  |  |  | BraA03g10982Z | BolC4g23544H | BnaA03G0147300ZS |
|  |  |  |  |  | BraA04g17848Z | BolC4g27444H | BnaC03G0171900ZS |
|  |  |  |  |  | BraA03g10983Z | BolC3g14389H | BnaA03G0147400ZS |
|  |  |  |  |  | BraA04g17849Z | BolC3g14391H | BnaC04G0500400ZS |
|  |  |  |  |  |  |  | BnaA05G0132000ZS |
|  |  |  |  |  |  |  | BnaA04G0196800ZS |
|  |  |  |  |  |  |  | BnaA04G0196700ZS |
|  |  |  |  |  |  |  | BnaC04G0500500ZS |
|  |  |  |  |  |  |  | BnaC04G0174600ZS |
|  |  |  | *4CL1* | AT1G51680 | BraA05g20849Z | BolC6g36477H | BnaC06G0113600ZS |
|  |  |  |  |  |  |  | BnaA05G0171400ZS |
|  |  |  | *4CL2* | AT3G21240 | BraA05g21635Z | BolC5g32971H | BnaC05G0371900ZS |
|  |  |  |  |  |  |  | BnaC05G0372000ZS |
|  |  |  |  |  |  |  | BnaA05G0345400ZS |
|  |  |  | *4CL3* | AT1G65060 | BraA07g30902Z | BolC6g38989H | BnaC06G0322800ZS |
|  |  |  |  |  |  |  | BnaA07G0281800ZS |
|  |  |  | *4CL5* | AT3G21230 | BraA03g13366Z | BolC5g32974H | BnaA05G0345800ZS |
|  |  |  |  |  | BraA05g21638Z | BolC5g32977H | BnaA03G0366500ZS |
|  |  |  |  |  | BraA05g21637Z | BolC5g32973H | BnaA05G0345500ZS |
|  |  |  |  |  | BraA05g21636Z | BolC3g17441H | BnaC03G0447200ZS |
|  |  |  |  |  | BraA03g13367Z | BolC3g17440H | BnaC05G0372100ZS |
|  |  |  |  |  |  |  | BnaC05G0372300ZS |
|  |  |  |  |  |  |  | BnaC05G0372800ZS |
|  |  |  |  |  |  |  | BnaA03G0366600ZS |
|  |  |  |  |  |  |  | BnaC05G0372600ZS |
|  |  |  |  |  |  |  | BnaC03G0447300ZS |
|  | Early |  | *CHS* | AT5G13930 | BraA02g08746Z | BolC1g00613H | BnaC01G0059700ZS |
|  | biosynthetic |  |  |  | BraA02g05198Z | BolC3g13229H | BnaC02G0468500ZS |
|  | genes |  |  |  | BraA02g08745Z | BolC9g53081H |  |
|  |  |  |  |  | BraA03g09960Z | BolC2g06596H |  |
|  |  |  |  |  | BraA09g39526Z | BolC2g11735H |  |
|  |  |  |  |  | BraA10g44510Z | BolC9g59153H |  |
|  |  |  |  |  |  | BolC2g11740H |  |
|  |  |  |  |  |  | BolC2g11734H |  |
|  |  |  |  |  |  | BolC2g11737H |  |
|  |  |  |  |  |  | BolC2g11530H |  |
|  |  |  | *CHI* | AT3G55120 | BraA03g15453Z | BolC6g37453H | BnaC08G0351800ZS |
|  |  |  |  |  | BraA07g29921Z | BolC8g50661H | BnaC08G0351900ZS |
|  |  |  |  |  | BraA09g40267Z | BolC7g46557H | BnaA09G0510300ZS |
|  |  |  |  |  | BraA09g40269Z | BolC8g50659H | BnaA07G0191700ZS |
|  |  |  |  |  |  |  | BnaA03G0551500ZS |
|  |  |  |  |  |  |  | BnaA09G0510400ZS |
|  |  |  |  |  |  |  | BnaC06G0193800ZS |
|  |  |  |  |  |  |  | BnaC07G0527900ZS |
|  |  |  | *F3H* | AT3G51240 | BraA09g39887Z | BolC7g45140H | Bnascaffold0027G0063600ZS |
|  |  |  |  |  | BraA09g40985Z | BolC4g25404H | BnaC08G0314600ZS |
|  |  |  |  |  | BraA03g13929Z | BolC8g50265H | BnaA04G0049500ZS |
|  |  |  |  |  |  |  | BnaA09G0576300ZS |
|  |  |  |  |  |  |  | BnaC07G0392300ZS |
|  |  |  |  |  |  |  | BnaA09G0476900ZS |
|  |  |  |  |  |  |  | BnaC08G0427800ZS |
|  |  |  |  |  |  |  | BnaA03G0419600ZS |
|  |  |  | *F3'H* | AT5G07990 | BraA10g44913Z | BolC9g59639H | BnaC09G0570900ZS |
|  |  |  |  |  |  |  | BnaA10G0256900ZS |
|  |  |  | *FLS1* | AT5G08640 | BraA09g36485Z | BolC9g53856H | BnaC08G0286600ZS |
|  |  |  |  |  | BraA10g44859Z | BolC4g27539H | BnaC03G0540900ZS |
|  |  |  |  |  | BraA06g25971Z | BolC9g59578H | BnaC04G0508000ZS |
|  |  |  |  |  | BraA06g25028Z | BolC3g18539H | BnaC09G0079000ZS |
|  |  |  |  |  |  |  | BnaA10G0251700ZS |
|  |  |  |  |  |  |  | BnaA06G0169300ZS |
|  |  |  |  |  |  |  | BnaA09G0085100ZS |
|  |  |  |  |  |  |  | BnaA06G0287200ZS |
|  |  |  |  |  |  |  | BnaC09G0565100ZS |
|  |  |  | *FLS2* | AT5G63580 |  |  |  |
|  |  |  | *FLS3* | AT5G63590 | BraA02g09210Z | BolC8g49953H | BnaA02G0404600ZS |
|  |  |  |  |  | BraA02g09211Z | BolC9g53855H | BnaC02G0537300ZS |
|  |  |  |  |  | BraA06g25972Z | BolC2g12355H | BnaC09G0078900ZS |
|  |  |  |  |  |  | BolC3g18538H | BnaC02G0537400ZS |
|  |  |  |  |  |  | BolC2g12354H | BnaA06G0287300ZS |
|  |  |  |  |  |  |  | BnaC03G0540700ZS |
|  |  |  | *FLS4* | AT5G63595 |  |  |  |
|  |  |  | *FLS5* | AT5G63600 |  |  |  |
|  |  |  | *FLS6* | AT5G43935 |  |  |  |
|  | Late biosynthetic |  | *DFR* | AT5G42800 | BraA09g37608Z | BolC9g55434H | BnaC02G0378400ZS |
|  | genes |  |  |  |  | BolC2g10369H | BnaC09G0215200ZS |
|  |  |  |  |  |  |  | BnaA09G0187400ZS |
|  |  |  | *ANS* | AT4G22880 | BraA01g01358Z | BolC1g01672H | BnaC01G0158400ZS |
|  |  |  |  |  |  | BolC7g45718H | BnaC07G0445600ZS |
|  |  |  |  |  |  |  | BnaA03G0469300ZS |
|  |  |  |  |  |  |  | BnaA01G0127300ZS |
|  |  |  | *UGT79B1* | AT5G54060 | BraA10g43226Z | BolC4g27012H | BnaC04G0462200ZS |
|  |  |  |  |  | BraA06g25298Z | BolC4g27007H | BnaC09G0362400ZS |
|  |  |  |  |  |  | BolC9g57287H | BnaA10G0103400ZS |
|  |  |  |  |  |  | BolC4g27004H | BnaC04G0462100ZS |
|  |  |  |  |  |  | BolC4g27005H | BnaA06G0190700ZS |
|  |  |  |  |  |  | BolC4g27010H |  |
|  |  |  |  |  |  | BolC4g27014H |  |
|  |  |  | *UGT75C1* | AT4G14090 | BraA08g33031Z | BolC8g48098H | BnaC08G0116200ZS |
|  |  |  |  |  |  |  | BnaA08G0082300ZS |
|  |  |  | *UGT78D2* | AT5G17050 | BraA02g05356Z | BolC2g06788H | BnaC02G0077700ZS |
|  |  |  |  |  |  |  | BnaA02G0067600ZS |
| Regulatory | Positive | R2R3-MYB | *MYB11* | AT3G62610 |  |  |  |
| genes | regulators | independent | *MYB12* | AT2G47460 | BraA03g11814Z | BolC4g28762H | BnaC04G0615400ZS |
| (transcription |  | regulatory |  |  | BraA05g19045Z | BolC3g15448H | BnaC04G0005700ZS |
| factor) |  | genes |  |  |  |  | BnaA03G0227800ZS |
|  |  |  |  |  |  |  | BnaC03G0268000ZS |
|  |  |  |  |  |  |  | BnaA05G0004800ZS |
|  |  |  | *MYB111* | AT5G49330 | BraA09g36114Z | BolC2g11793H | BnaA02G0366600ZS |
|  |  |  |  |  | BraA06g26754Z | BolC9g53440H | BnaA09G0051900ZS |
|  |  |  |  |  |  | BolC7g44513H | BnaA06G0357300ZS |
|  |  |  |  |  |  |  | BnaC09G0038300ZS |
|  |  |  |  |  |  |  | BnaC02G0491800ZS |
|  |  | Regulation | *MYB113* | AT1G66370 |  |  |  |
|  |  | by forming | *MYB114* | AT1G66380 |  |  |  |
|  |  | MBW | *PAP1* | AT1G56650 | BraA02g06422Z | BolC3g17587H | BnaC06G0328700ZS |
|  |  | complex |  |  | BraA03g13471Z | BolC6g39058H | BnaC02G0205500ZS |
|  |  |  |  |  |  | BolC2g08331H | BnaA07G0286900ZS |
|  |  |  |  |  |  |  | BnaA02G0160600ZS |
|  |  |  |  |  |  |  | BnaC03G0461400ZS |
|  |  |  |  |  |  |  | BnaC06G0328400ZS |
|  |  |  |  |  |  |  | BnaA03G0376600ZS |
|  |  |  | *PAP2* | AT1G66390 | BraA07g30963Z | BolC6g39061H | BnaC06G0329100ZS |
|  |  |  |  |  |  |  | BnaA07G0287000ZS |
|  |  |  | *bHLH* |  |  |  |  |
|  |  |  | *EGL3* | AT1G63650 | BraA09g37031Z | BolC8g52296H | BnaC09G0142800ZS |
|  |  |  |  |  | BraA09g39634Z | BolC7g42824H | BnaC08G0507300ZS |
|  |  |  |  |  | BraA09g37182Z | BolC9g54763H | BnaA09G0148900ZS |
|  |  |  |  |  |  | BolC9g54557H | BnaC07G0193400ZS |
|  |  |  |  |  |  | BolC7g41093H | BnaA09G0134400ZS |
|  |  |  |  |  |  |  | BnaC09G0162100ZS |
|  |  |  |  |  |  |  | BnaC04G0438300ZS |
|  |  |  |  |  |  |  | BnaC07G0060400ZS |
|  |  |  | *GL3* | AT5G41315 | BraA04g17090Z |  | BnaA04G0124900ZS |
|  |  |  |  |  |  |  | BnaC04G0412800ZS |
|  |  |  | *TT8* | AT4G09820 | BraA09g38474Z | BolC9g56525H | BnaA09G0260600ZS |
|  |  |  |  |  |  | BolC9g56526H | BnaC09G0307800ZS |
|  |  |  |  |  |  |  | BnaC09G0307700ZS |
|  |  | WD40 | *TTG1* | AT5G24520 | BraA06g26458Z | BolC2g12080H | BnaC07G0364700ZS |
|  |  |  |  |  | BraA02g08998Z | BolC7g44845H | BnaA06G0329200ZS |
|  |  |  |  |  | BraA02g08997Z | BolC2g12081H | BnaC02G0515200ZS |
|  | Negative | Single- repeat | *MYBL2* | AT1G71030 |  |  | BnaA02G0192400ZS |
|  | regulators | R3 MYB |  |  |  |  | BnaA07G0323000ZS |
|  |  |  |  |  |  |  | BnaC02G0255900ZS |
|  |  |  |  |  |  |  | BnaC06G0377700ZS |
|  |  |  | *CPC* | AT2G46410 |  |  |  |
|  |  | LATERAL | *LBD37* | AT5G67420 | BraA09g36585Z | BolC2g12512H | BnaC07G0203800ZS |
|  |  | ORGAN |  |  | BraA07g29372Z | BolC9g53985H | BnaC09G0091600ZS |
|  |  | BOUNDARY |  |  | BraA02g09343Z | BolC7g42944H | BnaC02G0552400ZS |
|  |  | DOMAIN |  |  |  |  | BnaA09G0094400ZS |
|  |  | (LBD) |  |  |  |  | BnaA02G0416500ZS |
|  |  |  |  |  |  |  | BnaA07G0140200ZS |
|  |  |  | *LBD38* | AT3G49940 | BraA03g13875Z | BolC7g45081H | BnaA03G0414400ZS |
|  |  |  |  |  | BraA09g39775Z | BolC8g50115H | BnaA09G0467700ZS |
|  |  |  |  |  |  | BolC1g02981H | BnaC01G0272100ZS |
|  |  |  |  |  |  |  | BnaC07G0386100ZS |
|  |  |  |  |  |  |  | BnaC08G0301200ZS |
|  |  |  | *LBD39* | AT4G37540 | BraA03g15584Z | BolC7g46659H |  |
|  |  |  |  |  | BraA01g00137Z | BolC1g00154H |  |
|  |  |  |  |  |  | BolC9g53933H |  |
| Transport |  |  | *TT19* | AT5G17220 | BraA10g44276Z | BolC9g58832H | BnaC02G0078600ZS |
| genes |  |  |  |  | BraA02g05365Z | BolC2g06795H | BnaC09G0492500ZS |
|  |  |  |  |  |  |  | BnaA10G0196600ZS |
|  |  |  |  |  |  |  | BnaA02G0068400ZS |
