# Supplementary material for: Whole-Genome Identification and Comparative Expression Analysis of Anthocyanin Biosynthetic Genes in Brassica napus
Source: Front Genet. 2021 Nov 18;12:764835. doi: 10.3389/fgene.2021.764835 (PMC8636775; doi:10.3389/fgene.2021.764835)
Supplement: Supplementary file 7 [file Table4.DOCX]

Table S4 The corresponding primers of qRT-PCR

| Gene name | Forward primer | Reverse primer |
| --- | --- | --- |
| BnaC01G0158400ZS-F | ATGCACATTGGAGATACGTT | TTAGCCGGAGACTCAACACT |
| BnaC09G0362400ZS-F | ATTCCGCTGATTGGATACCTG | CTTCCCGTCAATGATCTCTCG |
| BnaC07G0445600ZS-F | AAGCCACAGGAAAGATCCAA | CGTGGCTTCTATGTAATCACT |
| BnaA03G0469300ZS-F | CCAAAAGAATACATCCGTCCA | CATAGCAGCCTTCTTGAGC |
| BnaA01G0127300ZS-F | AGATCTCTCACTTTGGCCTA | ACTTCATTCTCTAGACGGTCA |
| BnaC09G0215200ZS-F | TCTCACGATGCGACGATTCT | ATTGTCATCACTGCCCGGAA |
| BnaC05G0016900ZS-F | AGCTAGGCTTGGTGAGTCTAAGTAT | AACCCAAGCACTGACATGTGG |
| BnaA06G0190700ZS-F | CTTGCGGTTGCAATGAACCAA | CTTCCTCACTAGAGGCGGTTTA |
| BnaA02G0068400ZS-F | ACTATGCGACCAAATACTCCA | CGTCACATTCTTCGCCTAACCTG |
| BnaA09G0187400ZS-F | ATCCAGAATATAACGTGCCTT | CAGATTTCAAATGTTCCGGTA |
| BnaA10G0103400ZS-F | TCCCCAAGAAAGCTCTAGACCA | ACGCAGAATTGTCTCTACCTC |
| BnaC09G0570900ZS-F | CCAATTTCGCTAGCCGACCAC | CGCATGAGTGTTCCAACCTCT |
| BnaC03G0171900ZS-F | ACGCTTCGACTAAGAATGGCTA | TTCTTCCAGCTCTCGGGGTT |
| β-actiin-F | CTTCCTCACGCTATCCTCCG | AGCCGTCTCCAGCTCTTGC |
